# Supplementary material for: Preparation of Highly Crystalline Silk Nanofibrils and Their Use in the Improvement of the Mechanical Properties of Silk Films
Source: Int J Mol Sci. 2022 Sep 26;23(19):11344. doi: 10.3390/ijms231911344 (PMC9570172; doi:10.3390/ijms231911344)
Supplement: Supplementary file 1 [file ijms-23-11344-s001.zip › ijms-1923227-supplementary.pdf]

Supporting Information

Preparation of Highly Crystalline Silk Nanofibrils and their Use in the Improvement of the Mechanical Properties of Silk Films

Ji Hye Lee[1], Bo Kyung Park[2] and In Chul Um[1]*

1 Department of Biofibers and Biomaterials Science, Kyungpook National University, Daegu 41566, Republic of Korea
2 Buildings and Transportation Science Division, Oak Ridge National Laboratory, One Bethel Valley Road, Oak Ridge, TN 37831, USA

* Correspondence: icum@knu.ac.kr

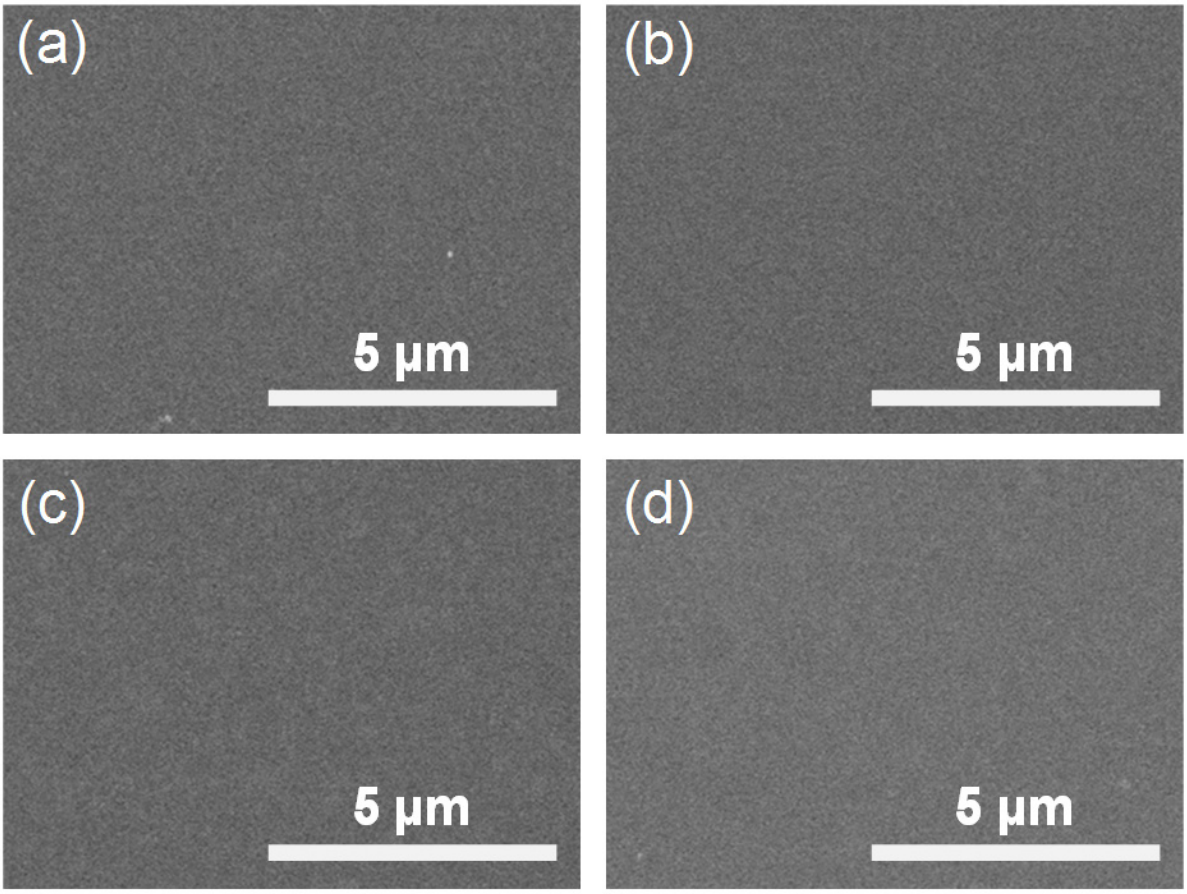

Figure S1. FE-SEM images of the surface of silk nanofibril/RSF composite films containing different amounts of silk nanofibrils; (a) 0%, (b) 5%, (c) 10%, and (d) 15%.
